# Long‐Term Performance of Two Systems for Automated Insulin Delivery in Adults With Type 1 Diabetes: An Observational Study

**DOI:** 10.1002/edm2.70043

**Published:** 2025-04-08

**Authors:** Sanne Fisker, Mia Christensen, Ermina Bach, Bo Martin Bibby, Klavs Würgler Hansen

**Affiliations:** ^1^ Steno Diabetes Center Aarhus Aarhus University Hospital Aarhus Denmark; ^2^ Medical Diagnostic Center Silkeborg Regional Hospital Silkeborg Denmark; ^3^ Medical Diagnostic Center Viborg Regional Hospital Viborg Denmark; ^4^ Section for Biostatistics, Department of Public Health Aarhus University Aarhus Denmark; ^5^ Department of Internal Medicine Aarhus University Aarhus Denmark

**Keywords:** automated insulin delivery, continuous glucose monitoring, glucose rate of change, glucose time in range, type 1 diabetes

## Abstract

**Aims:**

To compare glycaemic outcomes for two automated insulin delivery (AID) systems, the Tandem Control IQ (CIQ) and the MiniMed 780G (MM780G).

**Material and Methods:**

In this observational study, we evaluated 60 days of glycaemic data from 139 persons with type 1 diabetes (CIQ: 79 persons, MM780G: 60 persons), who had an active glucose sensor time ≥ 85%.

**Results:**

The time with AID was median 620 (IQR, 439–755) days for CIQ users and 509 (429–744) days for MM780G users (*p* = 0.26). The last HbA1c before initiation of AID was 59.7 mmol/mol in CIQ and 60.1 mmol/mol in MM780G (*p* = 0.88). The time with an active glucose sensor was higher for CIQ than MM780G (median 98.5 (97.4–98.0)% vs. 96.5 (94.9–97.0)%, *p* < 0.001). Time in range (TIR, glucose 3.9–10.0 mmol/L) was lower in CIQ than MM780G (mean 68.9% ± 11.4% vs. 73.7% ± 12.0%, *p* = 0.02) as was time in tight range (TITR) (glucose 3.9–7.8 mmol/L) (43.0% ± 12.2% vs. 48.4% ± 12.7%, *p* = 0.01). The difference in TIR (4.2 (95% CI 1.0–7.5)%, *p* = 0.01) and TITR (5.0 (1.4–8.6)%, *p* < 0.01) remained statistically significant in a multiple regression model controlling for various baseline variables. Time with an absolute rate of glucose change > 1.5 mmol/L/15 min was higher in CIQ than MM780G (9.4 (IQR, 7.2–13.3)% vs. 7.4 (5.2–10.4)%, *p* < 0.001).

**Conclusions:**

The CIQ system had a higher active glucose sensor time but a lower TIR, TITR, and a higher time with a rapid glucose rate of change than the MM780G system.

## Graphical abstract

This observational study of two algorithms for automated insulin delivery in type 1 diabetes showed differences in mean sensor glucose, time in range, glucose rate of change and the diurnal glucose profile.

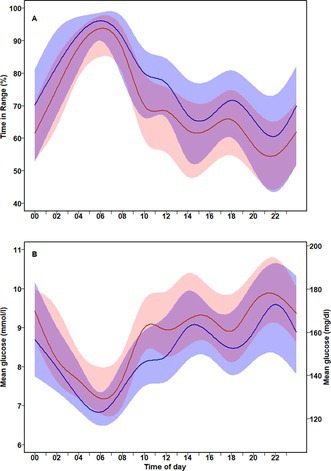

## 1 Introduction

Insulin pumps with automated insulin delivery (AID) have profoundly changed the glycaemic treatment of type 1 diabetes [1, 2, 3, 4]. While near normalisation of the glucose level is an obtainable goal for some persons, a sizeable fraction continues to experience suboptimal time in range (TIR) despite AID systems [5]. AID systems differ in glucose sensors, insulin delivery algorithms and pump settings [6, 7, 8]. Each has weaknesses and strengths, making it challenging for users and healthcare providers to choose the most suitable system. Factors such as daily variations in physical activity, technical skills, carbohydrate counting ability, IT literacy and the preference for tubed or tubeless pumps can influence the choice of the AID system. Many individuals lack a clear, optimal system recommendation even with these considerations.

Head‐to‐head studies of AID systems are rarely conducted [9] due to significant practical and economic obstacles to conducting randomised parallel studies. Crossover studies are even less feasible as each system requires extensive reinstruction. To our knowledge, no randomised study comparing two AID systems has yet been reported. Furthermore, the time frame for planning and conducting a randomised study is much longer than for upgrading the present AID system or the presentation of new systems. Two observational studies from Italy compared AID systems in children and adolescents after 1 month [10, 11] and after 12 months [12] with diverging results. An observational study from France reported differences in continuous glucose monitoring (CGM) data between two AID systems after 3–12 months, without a significant difference in HbA1c [13]. One recent prospective but non‐randomised study from Spain compared two AID systems in adults after 3 months and found only minor differences in glycaemic outcomes [14]. Lastly, two observational studies found a disconnection between changes in mean sensor glucose and HbA1c between two AID systems, suggesting an impact of different sensors [15, 16]. Otherwise, the assessment of the performance of different AID systems is typically based on single‐system ‘real‐world data’ from the upload of the pump and CGM data to a device‐specific software platform, with only limited access to self‐reported demographic data [8].

We aim to compare the long‐term CGM‐based performance of two widely used algorithms for AID systems in a retrospective observational study with access to verified clinical data.

## 2 Material and Methods

We contacted all persons with type 1 diabetes who had initiated AID before 31 December 2022 and who had used the AID system for at least 3 months either with the Tandem Control‐IQ (CIQ) insulin pump and Dexcom G6 sensor or the Medtronic MiniMed 780 G (MM780G) insulin pump with a Guardian 3 or 4 sensor.

Study participants were recruited from one university hospital (Steno Diabetes Center Aarhus, Denmark) and two regional hospitals in Denmark. We sent an electronic letter requesting informed consent to download pump and CGM data for 2 months from the Glooko (CIQ) or Carelink platform (MM780G) and permission to retrieve clinical data from their electronic health records. Persons who were pregnant or had received systemic steroid treatment in the study period were not invited.

For each person, we downloaded the most recent aggregate pump and insulin data along with all CGM data in the order consent was achieved, starting on 7 December 2022 and ending on 8 January 2024. Glucose data were truncated at 60 days and processed by the software package R. Active CGM time was calculated as the 5‐min periods with a glucose value divided by the total number of 5‐min periods in 60 days (17,280) multiplied by 100.

TIR was the number of glucose values from 3.9 to 10.0 mmol/L divided by the number of periods with a glucose value multiplied by 100. Time above range (TAR) (> 10 mmol/L), TAR level 2 (> 13.9 mmol/L), time in tight range (TITR) (3.9–7.8 mmol/L), time below range (TBR) (< 3.9 mmol/L) and TBR level 2 (< 3.0 mmol/L) were calculated similarly. The definition of TIRs follows the international consensus [17]. The glucose management indicator (GMI) was calculated as mean glucose (mmol/L) multiplied by 4.70587 plus 12.71 [18].

The glucose rate of change (RoC) was calculated in steps of 15 min as the slope of a regression line of glucose values from *t* = 0, *t* = 5, *t* = 10 and *t* = 15 min and given as mmol/L/15 min [19]. A rapid increase in glucose was defined as RoC > 1.5 mmol/L/15 min, and a rapid decrease in glucose was defined as RoC < −1.5 mmol/L/15 min. Time with rapid increase in glucose (TRC+) and decrease in glucose (TRC−) were calculated as previously described [20]. Each individual's total time with rapid change of glucose (TRC) is the sum of TRC+ and TRC−. The average absolute rate of change (AARC) was calculated as the mean of absolute RoC values [21, 22].

A hypoglycaemic event was defined as at least three consecutive 5‐min periods with glucose < 3.9 mmol/L, followed by at least three successive 5‐min periods with glucose ≥ 3.9 mmol/L [23]. A severe hypoglycaemic event was defined accordingly but with a glucose cut‐off value of 3.0 mmol/L. Rebound hyperglycaemia was defined by at least one glucose > 10 mmol/L in 120 min after a hypoglycaemic event [24].

The insulin/carbohydrate ratio (both systems) and insulin sensitivity (CIQ only) were calculated as a time‐weighted mean of the 24 h. If the person had more than one 24‐h profile, we chose ‘work‐day’ or similar to indicate the most commonly used profile.

The study was registered in the Central Denmark Region research database as no. 1‐16‐02‐388‐22.

### 2.1 Statistical Methods

Normal distribution was assessed by visual inspection of QQ plots. Normally distributed data are presented as mean ± standard deviation (SD), and data between groups were compared with Student's unpaired *t*‐test. Non‐normally distributed data were presented as median and inter‐quartile range, and groups were compared with Mann–Whitney *U* test. The precision of group differences was given as 95% CI of the mean difference or 95% CI of the median difference analysed by Hodges–Lehmann test. Proportions were compared with a *χ* ^2^ test. The contribution of the AID system to the variation of TIR and TITR was assessed by multiple linear regression with the following independent variables: the last HbA1c before AID, age, time with AID, clinical setting (regional or university hospital), diabetes duration, gender and body mass index (BMI). Statistical analysis was performed with SPSS ver. 20.0.

## 3 Results

A total of 208 persons were invited. A flow chart of the recruitment is shown in Table 1. Eight persons declined to share data, three persons did not use the AID pump anymore when approached, and one had emigrated. Of the remaining 196 persons, 32 never responded despite one e‐letter invitation followed by two phone calls. The fraction of non‐responders from the university hospital (30/121 = 25%) was higher than from regional hospitals (2/75 = 3%, *p* < 0.001). Four persons had no data available for upload.

**TABLE 1:** This is a flow diagram of the recruitment of study participants sorted by AID type (CIQ or MM780G) or hospital (university or regional).

	Total	CIQ	MM780G	University	Regional
Total	208	105	103	127	81
Emigrated	1		1		1
No pump anymore	3	2	1	1	2
	204	103	101		
Denied consent	8	4	4	5	3
No response	32	12	20	30	2
	164	87	77		
No data upload	4	2	2	2	2
	160	85	75		
Active sensor time < 85%	21	6	15	14	7
Study population	139	79	60	75	64

Of the remaining 160 persons (CIQ: 85 persons, MM780G: 75 persons), the active CGM time was higher in the CIQ group: median 98.4% (IQR 97.2%–98.7%) than in the MM780G group: 95.7% (89.3%–97.0%), median difference 2.3% (95% CI 1.8%–3.1%), *p* < 0.001. TIR was higher in the MM780G group (72.7% ± 12.3%) than in the CIQ group (68.7% ± 11.5%), the mean difference was 4.0% (0.3%–7.7%), *p* = 0.036. The same applied for TITR (47.9% ± 12.6% vs. 42.7% ± 12.0%), mean difference 5.1% (1.3%–9.0%), *p* < 0.01.

A total of 21 persons (CIQ: 6 persons, MM780G: 15 persons) had an active sensor time < 85% (median 64% (51%–80%)), leaving 139 persons with active sensor time ≥ 85% for comparison.

The clinical characteristics are shown in Table 2. The MM780G group was slightly older and had a marginally longer diabetes duration. Otherwise, the groups were comparable except for a statistically significantly higher fraction of predictive low glucose suspend systems (mostly MM640G) in the group shifting to MM780G. Fifty‐five persons in the MM780G group used the Guardian 4 sensor and five Guardian 3. Table 3 shows a detailed breakdown of insulin delivery and glucose measurement methods.

**TABLE 2:** Clinical characteristics.

	CIQ (*n* = 79)	MM780G (*n* = 60)	MM780G—CIQ (95% CI)	*p*
Male/female, numbers (%)	31/48 (39/61%)	27/33 (45/55%)		0.5
Age (years)	44.4 ± 14.6	48.7 ± 15.2	4.3 (−0.7 to 9.4)	0.09
Weight (kg)	81.0 ± 17.3	78.3 ± 16.1	−2.8 (−8.4 to 2.9)	0.34
BMI (kg/m^2^)	26.8 ± 4.9	26.0 ± 5.1	−0.8 (−2.5 to −0.8)	0.33
Diabetes duration (years)	25.4 ± 12.5	29.8 ± 14.6	4.4 (−0.2 to 8.9)	0.06
Last HbA1c before AID pump (%) (mmol/mol)	7.62 ± 1.13 59.7 ± 12.4	7.65 ± 1.07 60.1 ± 11.7	0.03 (−0.35 to 0.40) 0.3 (−3.8 to 4.4)	0.88
Time from last HbA1c to initiation of AID (days)	57 (13 to 106)	64 (28 to 125)	13 (−6 to 30)	0.16
Insulin delivery and glucose measurement before AID				
MDI + CGM or CSII + SMBG or CSII + UCGM, numbers (%)	31 (39%)	4 (7%)		< 0.001
CSII (LGS),^a^ numbers (%)	48 (61%)	56 (93%)		
Time with AID, (days)	620 (439 to 755)	509 (429 to 744)	−43 (−116 to 32)	0.26
University hospital/regional hospital, numbers (%)	42/37 (53%)/(47%)	33/27 (55%)/(45%)		0.87

*Note:* Numbers, mean ± SD or median (IQR).

Abbreviations: AID, automated insulin delivery; CGM, continuous glucose monitoring; CSII, continuous subcutaneous glucose infusion; LGS, low glucose suspension; MDI, multiple daily insulin injections; SMBG, self‐measurement of blood glucose; UCGM, unintegrated CGM.

^a^ This group includes 5 persons with the first‐generation hybrid closed loop MM670G (four switched to MM7780G and one to CIQ).

**TABLE 3:** A breakdown of the methods for insulin delivery and glucose measurement before converting to hybrid closed loop with the CIQ or MM780G systems.

	CIQ	MM780G
MDI, CGM	7	1
CSII, SMBG	6	1
CSII, UCGM	18	2
CSII, PLGS	47	52
CSII, (first‐generation advanced hybrid close loop = MM670G)	1	4

Abbreviations: CGM, continuous glucose monitoring; CSII, continuous subcutaneous glucose infusion; MDI, multiple daily insulin injections; PLGS, predictive low glucose suspension; SMBG, self‐measurement of blood glucose; UCGM, unintegrated CGM.

Insulin data and pump settings are shown in Table 4. Persons with the MM780G system had a statistically significantly higher fraction of total insulin as a bolus than those using the CIQ system. The active CGM time was higher for CIQ, but the time with automation mode was higher in MM780G. Some pump settings are non‐adjustable, such as the active insulin time (5 h) and glucose target (6.1 mmol/L, in the daytime without activity) in the CIQ system. The glucose target in the MM780G had three different settings. The median value (6.0 mmol/L) was very close to the target for CIQ. For the MM780G, insulin sensitivity is an integrated function in the algorithm. Importantly, the two systems had comparable insulin‐carbohydrate ratios.

**TABLE 4:** Insulin data and pump settings.

	CIQ (*n* = 79)	MM780G (*n* = 60)	MM780G—CIQ (95% CI)	*p*
Total insulin (U)	50.6 ± 23.6^a^	44.7 ± 23.7	−5.9 (−14.1 to 2.3)	0.16
Insulin (U/kg)	0.60 ± 0.20^a^	0.55 ± 0.20	−0.05 (−0.12 to 0.02)	0.15
Bolus/total insulin (%)	51.6 ± 10.4^a^	57.0 ± 7.0	5.4 (2.3 to 8.6)	0.001
User‐initiated bolus/total insulin (%)	NA	39.6 ± 12.7		NR
Autocorrection bolus/total insulin (%)	NA	17.4 ± 7.4		NR
Announced meals per day (numbers)	4.4 (3.3 to 5.4)^b^	4.9 (3.8 to 6.0)	0.6 (−0.1 to 1.2)	0.08
Reported carbohydrate per day (g)	146 ± 74^b^	158 ± 92	12 (−17 to 41)	0.41
Carbohydrate/insulin ratio (g/U)	9.8 ± 4.0^c^	10.4 ± 3.9	0.6 (−0.7 to 2.0)	0.37
Insulin sensitivity (mmol/L/U)	2.5 (1.6 to 3.1)^c^	NA		NA
Duration of active insulin (h)	5.0 (5.0 to 5.0)	2.0 (2.0 to 3.0)		NR
Target glucose (daytime, no exercise) mmol/L	From 6.3 to 8.9	6.0 (6.0 to 6.0)		NR
5.5 mmol/L, numbers (%)	NA	31 (52%)		
6.1 mmol/L	NA	16 (27%)		
6.7 mmol/L	NA	13 (22%)		
Active CGM time (%)	98.5 (97.4 to 98.0)	96.5 (94.9 to 97.0)	−1.9 (−2.3 to −1.4)	< 0.001
Automation mode (%)	97.0 (94.0 to 98.0)^a^	98.0 (96 to 99)	1.0 (0.0 to 2.0)	< 0.01
Low alarm setting (mmol/L)	4.0 ± 0.4	3.8 ± 0.4^d^	−0.2 (−0.3 to −0.1)	< 0.01
High alarm setting (mmol/L)	13.3 ± 2.1	13.4 ± 2.2^e^	0.1 (−0.7 to 0.8)	0.82

*Note:* Mean ± SD, median (IQR) or numbers.

Abbreviations: NA, not available; NR, not relevant.

^a^ (*n* = 72).

^b^ (*n* = 71).

^c^ (*n* = 77).

^d^ (*n* = 56).

^e^ (*n* = 52).

**FIGURE 1:**
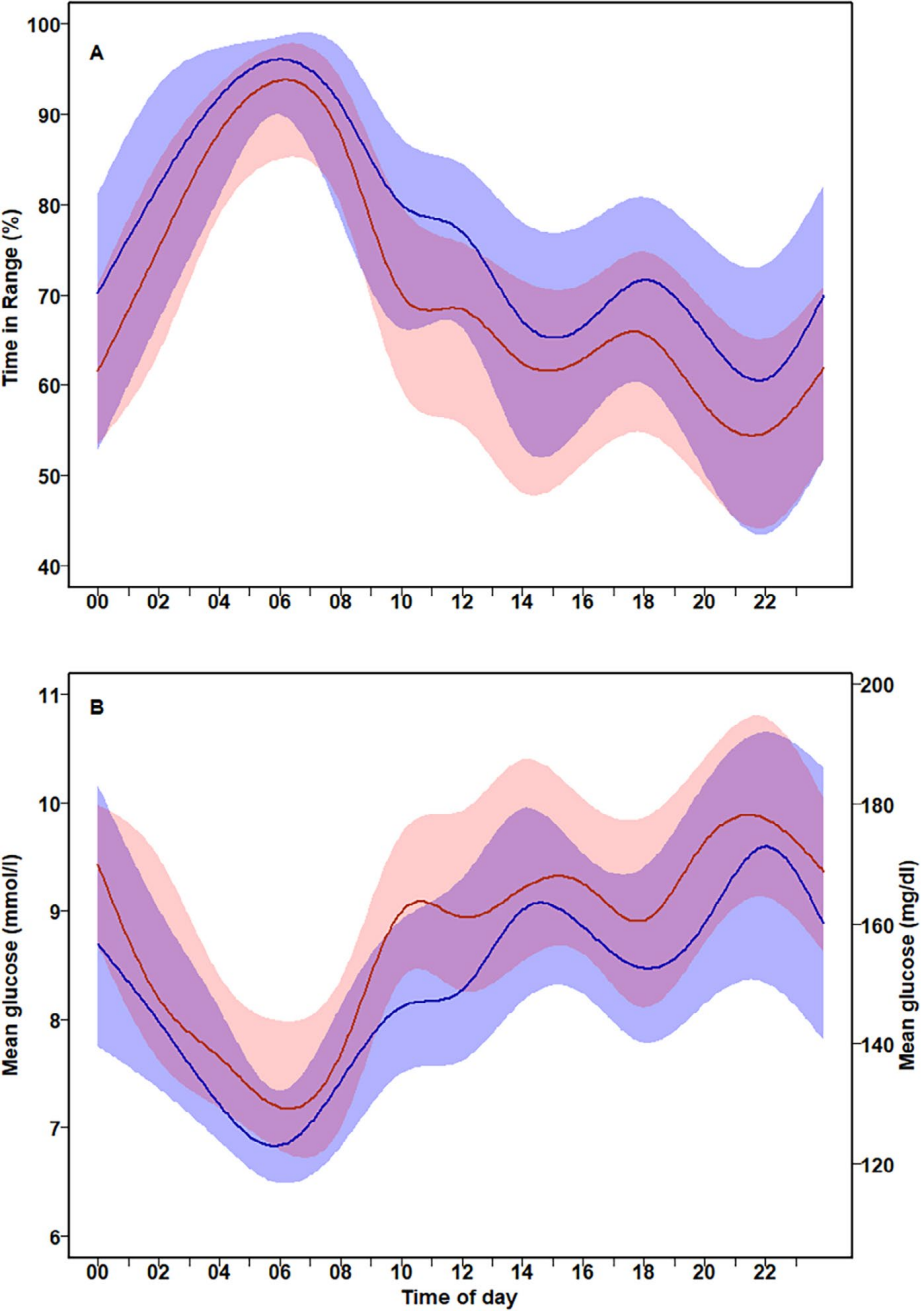
Twenty‐four hour variation of glucose time in range (3.9–10.0 mmol/L) (A) and glucose presented as median (IQR). Red colour for the CIQ system and blue for the MM780G system.

**FIGURE 2:**
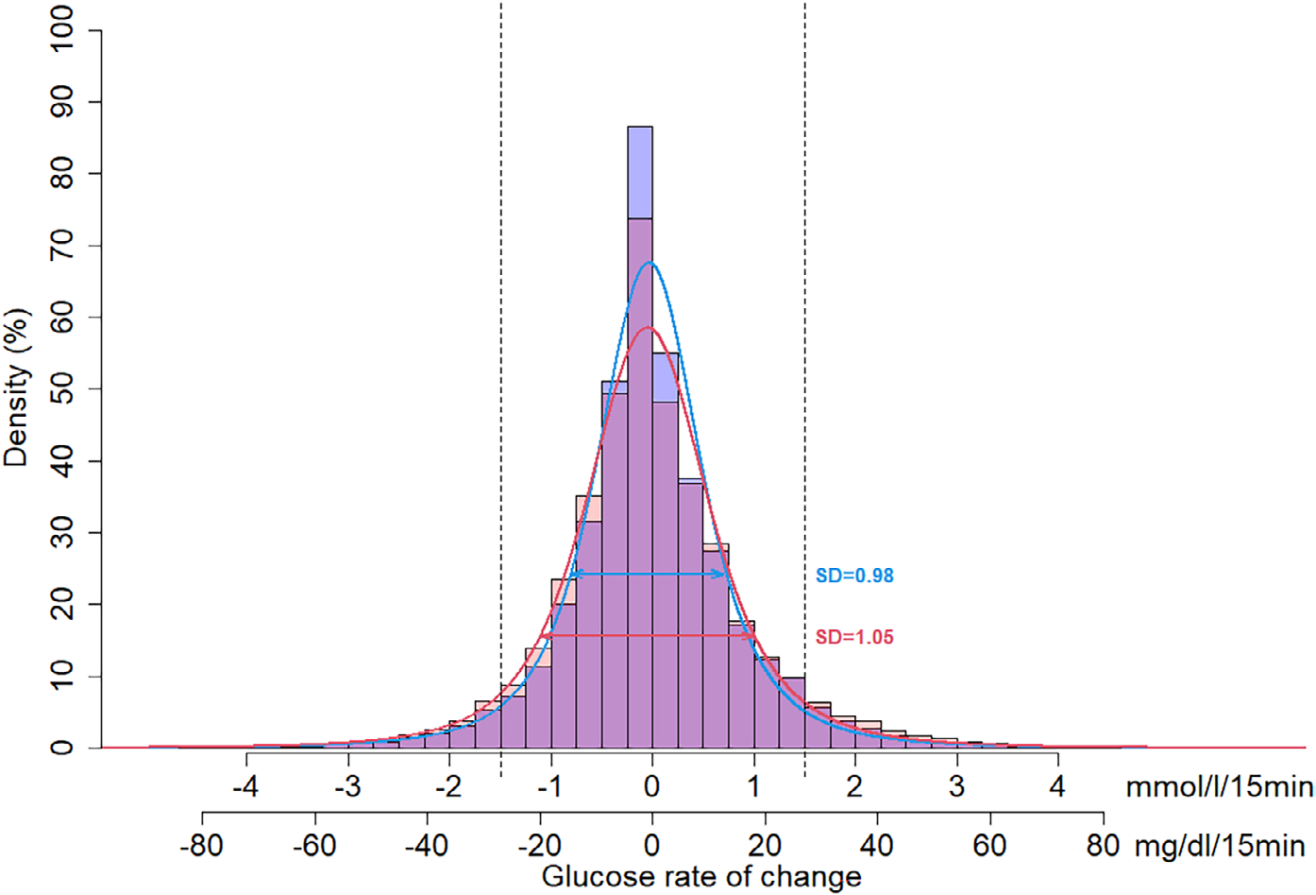
Histogram of the distribution of glucose rate of changes (RoC) with indication of SD for the t location scale distribution. For the CIQ system, the curve (red) was characterised by a location parameter −0.05 (mmol/L/15 min), SD 1.05 (mmol/L/15 min), shape parameter 0.63 and degrees of freedom 3.12. For the MM780G system (blue), the mean location parameter was −0.03 (mmol/L/15 min), SD 0.98 (mmol/L/15 min), shape parameter 0.54 and degrees of freedom 2.89. The vertical dotted lines represent RoC of ± 1.5 mmol/L/15 min.

The glycaemic metrics are shown in Table 5. The MM780G had a statistically significant lower TAR −4.8% (95% CI −8.8% to −0.6%) and a higher TIR 4.8% (0.8%–8.7%) and TITR 5.4% (1.2%–9.6%) than CIQ. The proportion of persons with TITR > 55% was 11% (9/79) for CIQ and 35% (21/60) for the MM780G group, with a difference in proportions of 24% (95% CI 10%–38%), *p* = 0.001. The diurnal variation of TIR and mean glucose is shown in Figure 1. The frequency of hypoglycaemic events per 14 days for the combined groups was a median of 4.9 (IQR: 2.3–9.1), and the frequency of rebound hyperglycaemic events was 2.9 (0.7–4.2). The fraction of all hypoglycaemic events leading to rebound hyperglycaemia was 45.1% (±20.7%), with no statistically significant differences between the systems (Table 5). The RoC values were *t*‐scale location distributed for both systems with different shapes (Figure 2). Glucose SD, CV, AARC and TRC were higher with the CoIQ than the MM780G system.

**TABLE 5:** Glycemic metrics.

	CIQ (*n* = 79)	MM780G (*n* = 60)	MM780G—CIQ (95% CI)	*p*
Glucose (mmol/L)	8.9 ± 1.1	8.5 ± 1.0	−0.4 (−0.8 to −0.1)	0.03
Glucose SD (mmol/L)	3.0 ± 0.6	2.8 ± 0.7	−0.3 (−0.5 to −0.03)	0.02
Glucose CV (%)	33.7 ± 5.1	32.3 ± 4.8	−1.5 (−3.1 to 0.2)	0.09
GMI (mmol/mol)	54.6 ± 5.0	52.7 ± 4.9	−1.9 (−3.6 to −0.2)	0.03
TAR level 2 (%)	6.6 (3.2 to 10.6)	3.7 (1.7 to 8.3)	−2.2 (−3.8 to −0.8)	< 0.01
TAR (%)	29.5 ± 11.8	24.8 ± 12.4	−4.8 (−8.8 to −0.6)	0.02
TIR (%)	68.9 ± 11.5	73.7 ± 12.0	4.8 (0.8 to 8.7)	0.02
TITR (%)	43.0 ± 12.2	48.4 ± 12.7	5.4 (1.2 to 9.6)	0.01
TBR (%)	1.2 (0.5 to 2.3)	1.1 (0.6 to 2.1)	0.0 (−0.4 to 0.3)	0.76
TBR (level 2) (%)	0.2 (0.1 to 0.4)	0.2 (0 to 0.4)	0.0 (−0.1 to 0.0)	0.33
Hypoglycemic events per 14 days (< 3.9 mmol/L)	5.6 (2.1 to 9.8)	4.7 (2.5 to 8.6)	−0.5 (−2.1 to 0.9)	0.47
Severe hypoglycaemic events per 14 days (< 3.0 mmol/L)	0.7 (0.2 to 2.1)	0.7 (0.2 to 1.6)	0.0 (−0.3 to 0.2)	0.77
Rebound hyperglycaemic events/14 days	2.6 (0.9 to 4.2)	2.1 (0.7 to 3.6)	0.4 (−1.1 to 0.2)	0.22
Rebound hyperglycaemic events/all hypoglycaemic events (%)	46.6 (± 20.4)	43.1 (± 21.1)	−3.6 (−10.7 to 3.5)	0.32
TRC (%)	9.4 (7.2 to 13.3)	7.4 (5.2 to 10.4)	−2.1 (−3.3 to −0.8)	0.002
TRC+ (%)	5.5 (4.2 to 7.5)	4.0 (3.1 to 6.0)	−1.4 (−2.2 to −0.8)	< 0.001
TRC− (%)	4.2 (2.8 to 5.9)	3.4 (2.4 to 4.8)	−0.7 (−1.2 to −0.01)	0.045
AARC (mmol/L/15 min)	0.67 ± 0.12	0.59 ± 0.1	−0.08 (−0.12 to −0.04)	< 0.001

*Note:* Mean ± SD or median (IQR).

Abbreviations: AARC, average absolute rate of change; GMI, glucose management indicator; TAR, time above range; TBR, time below range; TIR, time in range; TITR, time in tight range; TRC, time with rapid glucose change.

Multiple regression analysis was performed with TIR and TITR as the dependent variables and several clinical and demographic data as independent variables (*R* ^2^ = 0.41 and *R* ^2^ = 0.37, respectively, *p* < 0.001 for both). The type of AID system remained a statistically significant determinant of the variation in TIR and TITR when controlling for confounders (Table 6). In this model, TIR was 4.2% (95% CI 1.0%–7.5%) and TITR 5.0% (1.4%–8.6%) higher for MM780G than for CIQ (*p* = 0.01 and *p* < 0.01, respectively).

**TABLE 6:** Multiple regression with TIR and TITR as dependent variables and independent variables as listed (*R* ^2^ = 0.41 and *R* ^2^ = 0.37, respectively, *p* < 0.001 for both).

Independent variables	Dependent variable: TIR (%)		Dependent variable: TITR (%)	
	Beta coefficient (95% CI)	*p*	Beta coefficient (95% CI)	*p*
AID system: (CIQ = 0, MM780G = 1)	4.2 (1.0 to 7.5)	0.01	5.0 (1.4 to 8.6)	< 0.01
Last HbA1c before AID: (mmol/mol)	−0.5 (−0.6 to 0.4)	< 0.001	−0.5 (−0.7 to 0.4)	< 0.001
Age: (years)	0.19 (0.03 to 0.34)	0.02	0.14 (−0.04 to 0.31)	0.12
Time with AID (months)	0.2 (−0.1 to 0.5)	0.17	0.2 (−0.1 to 0.5)	0.27
Clinical setting: (regional = 0, university = 1)	0.8 (−2.6 to 4.2)	0.64	1.7 (−2.1 to 5.5)	0.37
Diabetes duration: (years)	0.03 (− 0.14 to 0.20)	0.73	0.02 (−0.17 to 0.21)	0.83
Gender: (female = 0, male = 1)	1.2 (2.0 to 4.5)	0.47	1.5 (−2.1 to 5.1)	0.41
BMI: (kg/m^2^)	0.07 (−0.25 to 0.39)	0.67	0.01 (−0.34 to 0.37)	0.94
Constant	82.6 (68.3 to 96.8)	0.001	61.8 (46.0 to 77.5)	< 0.001

## 4 Discussion

In this study, we demonstrate differences in CGM data between two AID systems and highlight the importance of discussing the study's limitations and clinical relevance.

### 4.1 Comparison of CGM Data

The users of the MM780G maintained a higher TIR and TITR than CIQ, with fewer rapid glucose fluctuations and lower CV. We suggest that TRC could be used as a benchmark to compare the capacity of AID systems to maintain glucose homeostasis [20]. As expected, the most recent HbA1c level before initiating AID significantly predicted the achieved TIR in the multiple regression analysis [25].

The two systems differ in their approach to correcting rising glucose levels. The MM780G increases basal insulin to the maximal level, followed by autocorrection boluses every 5 min. In contrast, the CIQ system also increases basal insulin but delivers 60% of a correction bolus every hour if glucose is predicted to exceed 10.0 mmol/L, which may result in more rapid glucose changes. The different approaches to correcting rising glucose levels may partly explain the higher TIR in the postprandial period in MM780G noticed in the diurnal curve. Additionally, the number of declared meals per day tended to be higher in individuals using the MM780G, which could further contribute to a higher TIR [25]. Another difference between the systems is the target glucose setting. The MM780G system allows for three different target options, with the lowest at 5.5 mmol/L. In contrast, the CIQ system has a fixed target for daytime and regular physical activity of 6.1 mmol/L for autocorrection and 6.3–8.9 mmol/L for basal insulin (6.3–6.7 mmol/L at night). In this study, the median target glucose for the MM780G system was 6.0 mmol/L. The close alignment in TIR and glucose during the early morning suggests that these slight differences in target settings are unlikely to account for the observed outcomes.

TITR is a new, more ambitious treatment goal launched in the era of AID systems. Emerging data associate TIR and TITR with diabetic complications [26, 27]. The proposed optimal for TITR has been > 45% or even > 55% [28, 29]. Notably, a significantly larger proportion of persons with the MM780G than the CIQ achieved a TITR > 55%. Furthermore, the difference in TIR between the two systems was driven primarily by differences in TITR rather than by variations in the glucose range from 7.8 to 10.0 mmol/L. This is in concert with cross‐sectional studies reporting a higher TITR/TIR ratio when TIR is increasing [15, 30].

### 4.2 Consequences of Comparing CGM From Different Sensors

Comparison of two AID systems based on CGM data derived from different sensor technologies can be flawed and must be interpreted cautiously [31, 32]. It has been suggested that the higher TIR achieved with the MM780G than with the CIQ system may be an artefact of sensor performance because HbA1c levels were similar [13]. It is essential to recognise that the two AID algorithms in our study were assessed based on CGM data recorded by their respective sensors, which may affect CGM‐based metrics [33]. We found higher mean sensor glucose in CIQ than MM780G, which may be explained solely by a systematic variation between the sensors. We have recently discussed how to compare the algorithms for AID systems using different sensors [34].

A small‐scale study in which participants used the two sensors simultaneously gave some support to this theory [35]. Still, the insulin delivery algorithm, as a data‐engineering product, can be evaluated in its own right from the sensor data that served as input during its development. Lastly, we report lower glucose SD, CV, AARC and TRC in MM780G, less likely to be affected by sensor performance.

### 4.3 Clinical Relevance

The mean difference in TIR and TITR between the two systems was close to 5%, considered clinically relevant for developing diabetic retinopathy [26]. The international consensus suggests that a difference ≥ 5% in TIR is clinically meaningful for individuals, and a ≥ 3% difference is significant for treatment groups [17]. Our results should be interpreted with caution as the lower limit of the 95% confidence interval is around 1%. Furthermore, the difference in TIR is based on different sensors and may not be valid if sensor data were standardised [33]. It is unknown if high glucose variation or high glucose rate of chance per se is associated with endothelial damage, and the clinical significance of the differences found in this study cannot be evaluated.

### 4.4 Rebound Hyperglycaemia

The frequency of hypoglycaemic and rebound hyperglycaemic events was comparable between the two AID systems. Nearly half of all hypoglycaemic episodes were followed by rebound hyperglycaemia, in contrast to about one‐third in populations using an unintegrated CGM system [24, 36]. This may be attributed to overcompensation with carbohydrate intake combined with insulin suspension [37, 38].

### 4.5 Active CGM Time and Time in Automation Mode

Glycemic metrics for AID systems only account for periods when the sensor is active. For patients with low active sensor usage, glycaemic metrics may not necessarily reflect glycaemic control during times without sensor data or automation mode. Evaluating AID systems requires comparable and sufficiently high active sensor time, which is defined in this study as ≥ 85%. In our cohort, 86.9% of the persons had an active CGM time > 85%, which comes close to the fraction of 88.5% reported in a French real‐world study with the MM780G system [39]. Even for this cut‐off, the active sensor time was slightly but statistically higher for the CIQ system. The difference in active CGM time between the two systems cannot fully be explained by the impact of warm‐up time (2 h) with an interval of 10 days for the Dexcom G6 sensor and 7 days for the Guardian sensor or the time for recharging the Guardian sensor. Notably, the MM780G group still showed higher TIR and TITR, with comparable confidence intervals for the differences, even when all patients were included, irrespective of their actual active CGM time.

### 4.6 Limitations

The primary limitations are the retrospective observational study design, the lack of follow‐up HbA1c and using two different sensors. The choice of the AID system was made jointly by the user and the healthcare professional. While the AID system remained a significant determinant of TIR after controlling for several clinical variables, some personal characteristics or unknown confounders may influence the preference or recommendation for a particular pump. Without randomisation, we cannot ensure that the two groups are comparable.

The proportion of persons who either declined to share data or did not respond to our study invitation raises concerns about potential selection bias. Non‐responders may have various reasons for not engaging, including reduced diabetes self‐care or reluctance to share data if glycaemic control is suboptimal. However, we do not believe this to be the case because nearly all non‐responders were from a university hospital. These individuals are more likely to receive numerous e‐letters with study invitations and questionnaires, leading to some study fatigue and a lower response rate.

Participants were initiated on their AID system by company instructors and followed up by trained nurses. As healthcare providers manage various AID systems, we cannot exclude that a healthcare provider subspecialised in a given system could achieve better outcomes through more precise pump setting adjustments. The MM780G system may have yielded improved results if the recommended optimal pump setting for active CGM time and target glucose had been consistently applied [25]. Also, the results for the CIQ may have been improved if a lower insulin sensitivity factor had been applied. In this sense, our results reflect a real world rather than an ideal situation.

We acknowledge that the initial HbA1c was analysed for clinical use only and was not perfectly timed to represent glycaemic control before AID.

### 4.7 New Studies Comparing CIQ and MM780G

Our finding of a TIR of 68.9% in the CIQ and 73.7% in the MM780G system in adults aligns with data from very large databases [8]. A recent cross‐sectional study in children and adolescents described lower TIR (68.2% vs. 75.2%) but lower HbA1c (6.7% vs. 6.9%) in CIQ compared with MM780G [15]. A Swedish study found a similar reduction in TIR after initiating AID with CIQ or MM780G but a higher reduction of HbA1c at follow‐up in CIQ users. This dissociation of CGM and HbA1c results underscores the inherent problems of using CGM data from different sensors [32].

A prospective observational study from Barcelona, which followed 75 persons using the CIQ and 75 persons using the MM780G system for 3 months, found a small and non‐significant difference in TIR between the two systems, with a TIR of 75.0% for the CIQ and 77.4% for the MM780G [14] Several differences exist between the two studies. The Spanish study was also non‐randomised but had a prospective design. We reported TIR and active CGM time over 60 days compared to 14 days for the Spanish study. Additionally, we excluded persons with an active CGM time < 85%, while the Spanish study included all participants but did not report active CGM time. The Spanish study included persons ≥ 14 years old, while our study focused exclusively on adults. However, none of these differences fully explain the different outcomes of the two studies. In the present study, the mean follow‐up since initiating AID was more than 1½ years. It can be hypothesised that an initial high motivation for persons who knew they were participating in a 3‐month study may fade away in the following years. This theory is not supported by reports of consistent improvement in TIR over 3 years for both AID systems [40, 41].

### 4.8 Strengths

Our study has relatively few participants, but unlike self‐reported demographic data from large software platforms, we combined CGM and pump data with verified clinical data, including weight, diabetes duration and baseline HbA1c. The same healthcare professionals followed the users of both AID systems in the same diabetes centres. Furthermore, the glycaemic metrics evaluated over a long period (60 days) were calculated using a consistent method applied directly to the raw glucose data in contrast to metrics derived from company‐specific software platforms. Finally, we present detailed information on the pump settings to clarify our findings further.

## 5 Conclusions

The active CGM time was higher with the sensor connected to the CIQ system, but the MM780G algorithm demonstrated lower glucose excursions and a more favourable diurnal glycaemic profile.

## Author Contributions

**Sanne Fisker:** conceptualization (equal), data curation (supporting), investigation (equal), methodology (equal), supervision (equal), validation (equal), writing – original draft (equal), writing – review and editing (equal). **Mia Christensen:** conceptualization (supporting), data curation (equal), investigation (equal), methodology (equal), project administration (equal), writing – review and editing (equal). **Ermina Bach:** conceptualization (equal), data curation (equal), investigation (equal), methodology (equal), supervision (equal), validation (equal), writing – review and editing (equal). **Bo Martin Bibby:** data curation (equal), formal analysis (equal), methodology (equal), software (equal), supervision (equal), validation (equal), visualization (equal), writing – review and editing (equal). **Klavs Würgler Hansen:** conceptualization (lead), data curation (lead), formal analysis (lead), funding acquisition (lead), investigation (equal), methodology (lead), project administration (lead), resources (lead), software (equal), supervision (lead), validation (lead), visualization (equal), writing – original draft (equal), writing – review and editing (lead).

## Conflicts of Interest

K.W.H. has received a grant from Abbott Diabetes Care and Novo Nordisk for an investigator‐initiated study.

## Supporting information


Data S1 (STROBE Checklist).

## Data Availability

The data that support the findings of this study are available from the corresponding author upon reasonable request.
