# Supplementary material for: Long‐Term Performance of Two Systems for Automated Insulin Delivery in Adults With Type 1 Diabetes: An Observational Study
Source: Endocrinol Diabetes Metab. 2025 Apr 8;8(3):e70043. doi: 10.1002/edm2.70043 (PMC11977919; doi:10.1002/edm2.70043)
Supplement: Supplementary file 1 — Data S1 (STROBE Checklist). [file EDM2-8-e70043-s001.docx]

STROBE Statement—Checklist of items that should be included in reports of ***cross-sectional studies***

**Item**

**No Recommendation**

| **Title and abstract** | 1 | (*a*) Indicate the study’s design with a commonly used term in the title or the abstract  “Title: Long-term performance of two advanced hybrid closed loop insulin delivery systems in adults with type 1 diabetes: An observational study.”  "Abstract: In this observational study…"  (*b*) Provide in the abstract an informative and balanced summary of what was done and what was found  Done |
| --- | --- | --- |
| **Introduction** |  |  |
| Background/rationale | 2 | Explain the scientific background and rationale for the investigation being reported  Done |
| Objectives | 3 | State specific objectives, including any prespecified hypotheses  "Introduction: We aim to compare the long-term CGM performance of two common AHCL systems in a retrospective observational study with access to verified clinical data." |
| **Methods** |  |  |
| Study design | 4 | Present key elements of study design early in the paper  "Research Design and Methods  We contacted all persons with type 1 diabetes who had initiated AHCL before 31 December 2022 and who had used the AHCL system for at least 3 months either with the Tandem Control-IQ (CoIQ) insulin pump and Dexcom G6 sensor, or the Medtronic MiniMed 780 G (MM780G) insulin pump with a Guardian 3 or 4 sensor “ |
| Setting | 5 | Describe the setting, locations, and relevant dates, including periods of recruitment, exposure, follow-up, and data collection  " Research Design and Methods  Study participants were recruited from one university hospital (Steno Diabetes Center Aarhus) and two regional hospitals in Denmark. We send an electronic letter requesting informed consent to download pump and CGM data for two months from the Glooko (CoIQ) or Carelink platform (MM780G) and to retrieve clinical data from their electronic health record. Persons who were pregnant or had received systemic steroid treatment in the study period were not invited.  For each person we downloaded the last available pump and CGM data in the sequence we achieved individual consent starting from 7 December 2022 and ending on 8 January 2024." |
| Participants | 6 | (*a*) Give the eligibility criteria and the sources and methods of selection of participants  "Results:  A total of 208 persons were invited. A flow diagram of the recruitment is shown in Supplemental Table S1." |
| Variables | 7 | Clearly define all outcomes, exposures, predictors, potential confounders, and effect modifiers. Give diagnostic criteria, if applicable  We mention time in ranges, time with rapid change of glucose, and the definition of hypoglycemic events and rebound hyperglycemic events.  Confounders were addressed in the multiple regression analysis with the following confounding variables: the last HbA1c before AHCL, age, time with AHCL, clinical setting (regional or university hospital), diabetes duration, gender and BMI. |
| Data sources/ measurement | 8* | For each variable of interest, give sources of data and details of methods of assessment (measurement). Describe comparability of assessment methods if there is more than one group  " Research Design and Methods:  We send an electronic letter requesting informed consent to download pump and CGM data for two months from the Glooko (CoIQ) or Carelink platform (MM780G) and to retrieve clinical data from their electronic health record.  For each person we downloaded the last available pump and CGM data in the sequence we achieved individual consent starting from 7 December 2022 and ending on 8 January 2024."  “Discussion”:  Comparison of two AHCL systems based on CGM data derived from different sensor technology can be flawed and must be interpreted with caution(31, 32). It has been suggested that the higher TIR achieved with the MM780G than with the CIQ system may be an artefact of sensor performance because HbA1c levels were similar(13). It is important to recognize that the two ACHCL algorithms in our study were assessed based on CGM data recorded by their respective sensors which may affect CGM based metrics(33). We found higher mean sensor glucose in CIQ than in MM780G, which may be explained solely by a systematic variation between the sensors.  A small-scale study in which participants used the two sensors simultaneously give some support to this theory(34). However, in that case it is puzzling that the difference in mean glucose varied over 24-hours. Still, the insulin delivery algorithm as a dataengineering product, can be evaluated in its own right from the sensor data that served as input during its development. |
| Bias | 9 | Describe any efforts to address potential sources of bias  Detailed discussion of bias is given including the following:  “The proportion of persons who either declined to share data or did not respond to our study invitation raises concerns about potential selection bias. Non-responders may have various reasons for not engaging, including reduced diabetes self-care or reluctance to share data if the glycemic control is suboptimal. However, we do not believe this to be the case because nearly all non-responders were from a university hospital. It is more likely that these individuals receive numerous e-letters with study invitations and questionnaires, leading to some study fatigue and a lower response rate. “ |
| Study size | 10 | Explain how the study size was arrived at  "Research Design and Methods  We contacted all persons with type 1 diabetes who have initiated AHCL before 31 December 2022 with either Tandem Control IQ (CoIQ) insulin pump and Dexcom G6 for CGM or Minimed 780 G (MM780G) insulin pump with a Guardian 3 or 4 CGM, who have used the AHCL system for at least 3 months." |
| Quantitative variables | 11 | Explain how quantitative variables were handled in the analyses. If applicable, describe which groupings were chosen and why  Please see the "Methods" section including "Statistical Analysis" |

Statistical methods 12 (*a*) Describe all statistical methods, including those used to control for confounding

I refer to "Statistical analysis" and the multiple regression (Supplementary Table S3) analysis to control for confounders.

1. Describe any methods used to examine subgroups and interactions: Se "Statistical analysis" section
2. Explain how missing data were addressed: Se flow diagram Supplementary Table S1
3. If applicable, describe analytical methods taking account of sampling strategy: Not relevant in this context

|  |  | (*e*) Describe any sensitivity analyses: Not relevant |
| --- | --- | --- |
| **Results** |  |  |

| Descriptive data | 14* | 1. Give characteristics of study participants (eg demographic, clinical, social) and information on exposures and potential confounders: See Table 1 and 2. 2. Indicate number of participants with missing data for each variable of interest: This is indicated in detail in the footnote to every Table. |
| --- | --- | --- |
| Outcome data | 15* | Report numbers of outcome events or summary measures: See Table 5 (hypoglycemic events and glycemic metrics) |
| Main results | 16 | (*a*) Give unadjusted estimates and, if applicable, confounder-adjusted estimates and their precision (eg, 95% confidence interval). Make clear which confounders were adjusted for and why they were included  Both adjusted and unadjusted estimates are given for the TIR and TTR difference between the two systems for insulin delivery. The 95 % CI interval for all group differences of continuous variables is given in a specific column in Tables 2,4 and 5.. |

Participants 13* (a) Report numbers of individuals at each stage of study—eg numbers potentially eligible, examined for eligibility, confirmed eligible, included in the study, completing follow-up, and analysed

Please see first section in "Results" and Table 1 and 2.

(

b) Give reasons for non-participation at each stage : See c)

(

c) Consider use of a flow diagram: See flow diagram Table 1.

(*b*) Report category boundaries when continuous variables were categorized:

Done for active CGM time.

|  |  | (*c*) If relevant, consider translating estimates of relative risk into absolute risk for a meaningful time period: Not relevant |
| --- | --- | --- |
| Other analyses | 17 | Report other analyses done—eg analyses of subgroups and interactions, and sensitivity analyses:  We have not analyzed subgroups |

| **Discussion** |  |
| --- | --- |
| Key results 18 | Summarise key results with reference to study objectives: Check |
| Limitations 19 | Discuss the limitations of the study, taking into account sources of potential bias or imprecision. Discuss both direction and magnitude of any potential bias  Very detailed discussion of limitations can be found in the "Limitations" section and in the “Clinical relevance” section including the following sentences:  “The mean difference in TIR and TITR between the two systems was close to 5%, which is considered clinically relevant for the development of diabetic retinopathy(26). The international consensus suggests that a difference ≥ 5% in TIR is clinically meaningful for individuals, and a ≥3% difference is significant for treatment groups(17). Our results should be interpreted with caution as the lower limit of the 95% confidence interval is around 1%.”  ” |
| Interpretation 20 | Give a cautious overall interpretation of results considering objectives, limitations, multiplicity of analyses, results from similar studies, and other relevant evidence  Yes: se 19) |
| Generalisability 21 ” | Discuss the generalisability (external validity) of the study results  "As healthcare providers manage various AHCL systems, we cannot exclude that a healthcare provider subspecialized in a given system could achieve better outcomes through more precise pump setting adjustments. It is also arguable that the MM780G system may have yielded improved results if the recommended optimal pump setting for active CGM time and target glucose had been consistently applied(25). Also, the results for the CIQ may have been improved if a lower insulin sensitivity factor had been applied. In this sense, our results reflect a real world rather than an ideal situation.” |
| **Other information** |  |

Funding 22 Give the source of funding and the role of the funders for the present study and, if applicable, for the original study on which the present article is based

Done

*Give information separately for exposed and unexposed groups.
